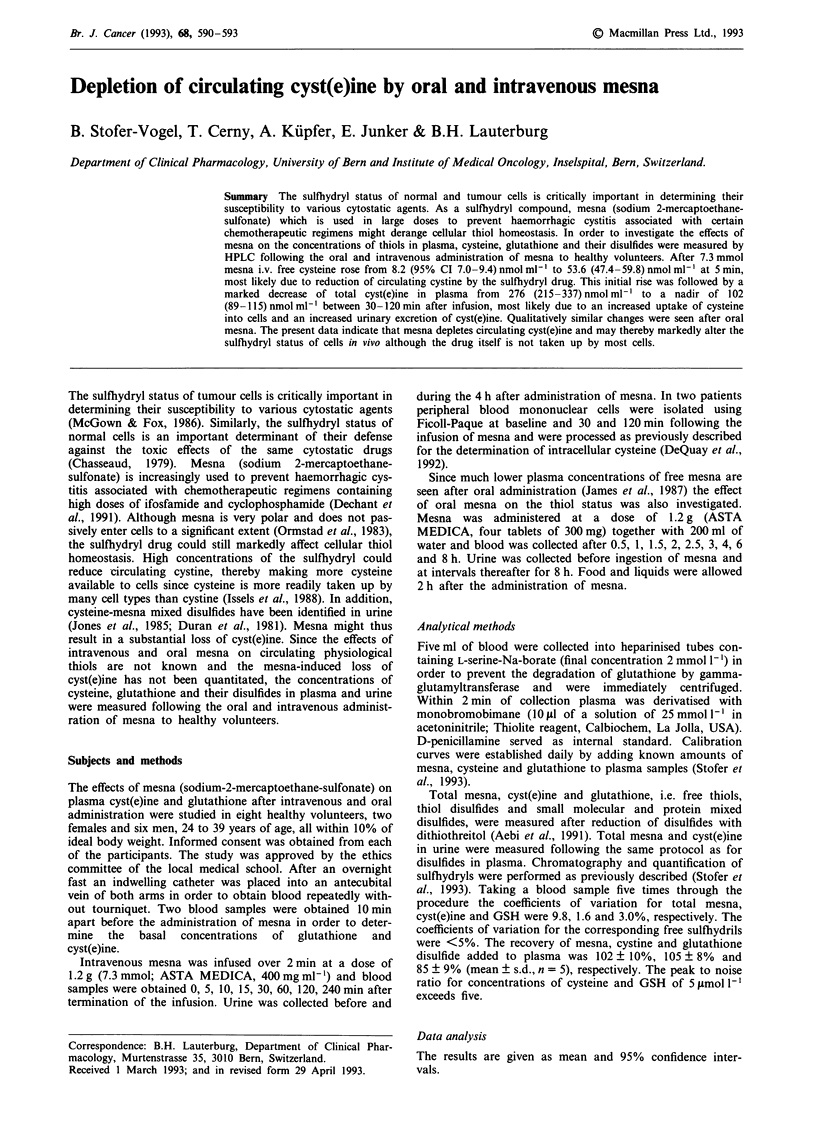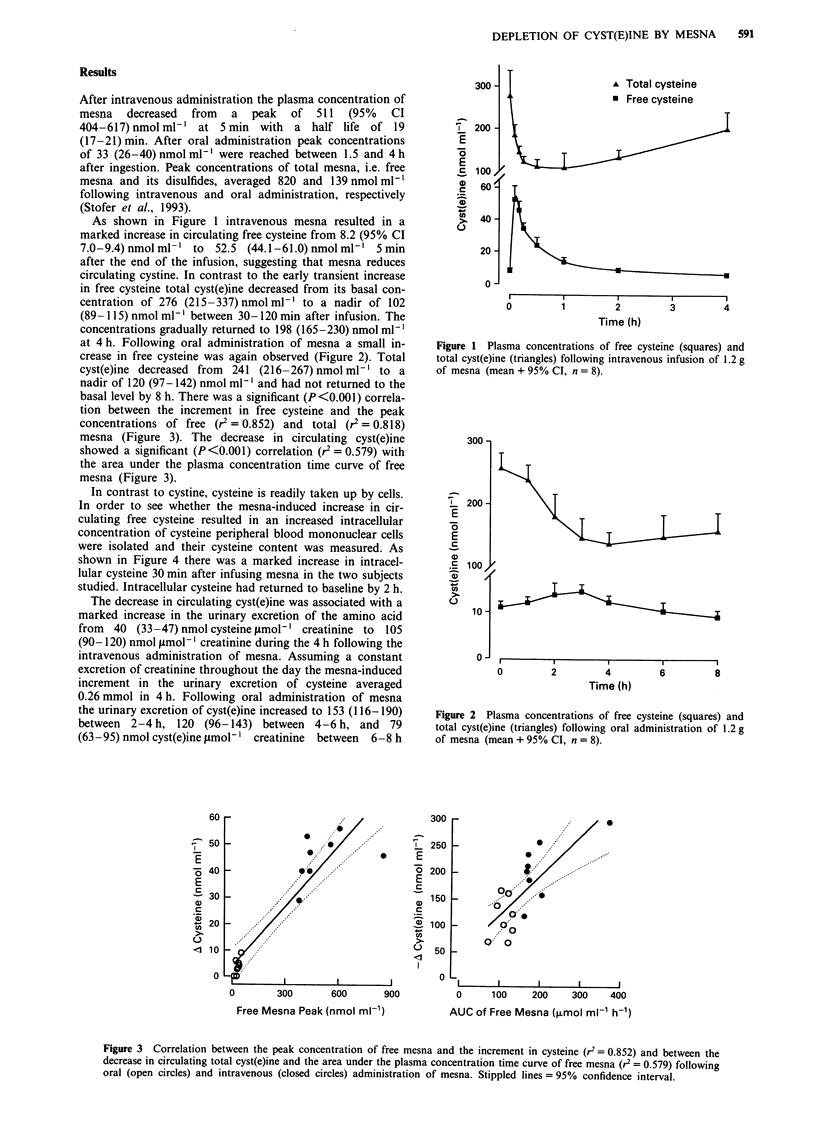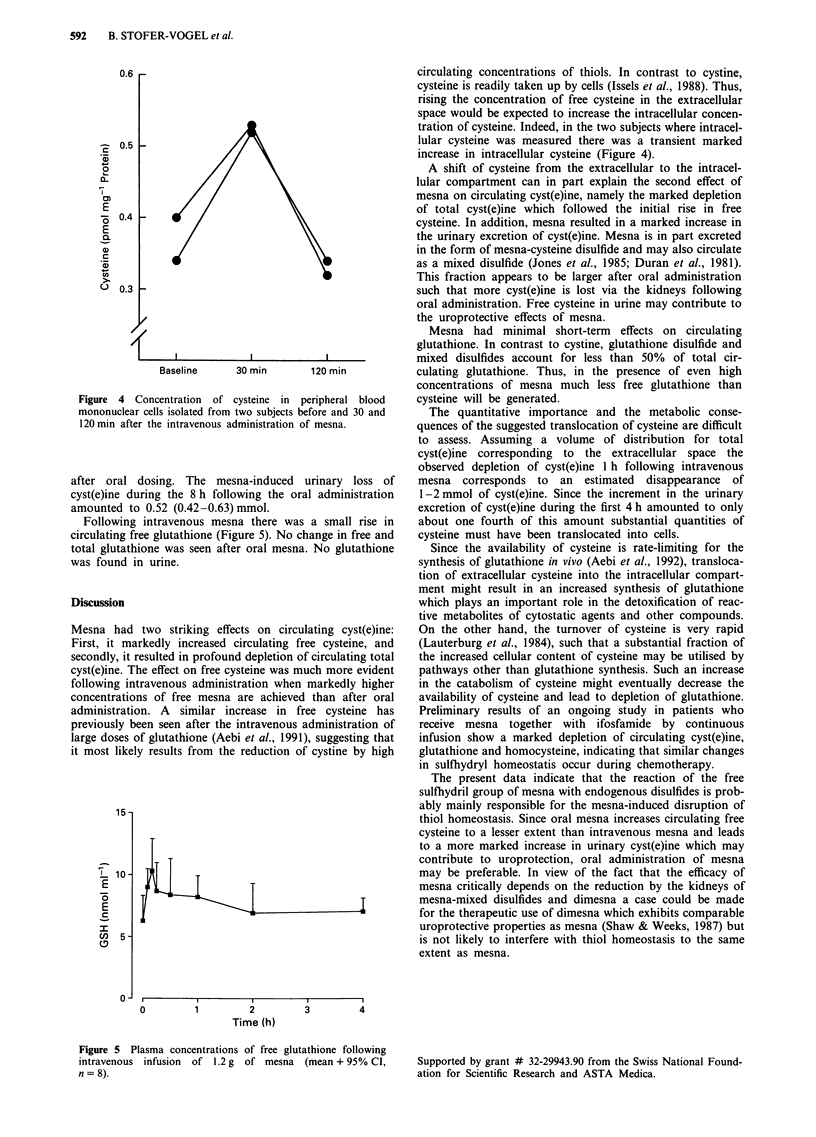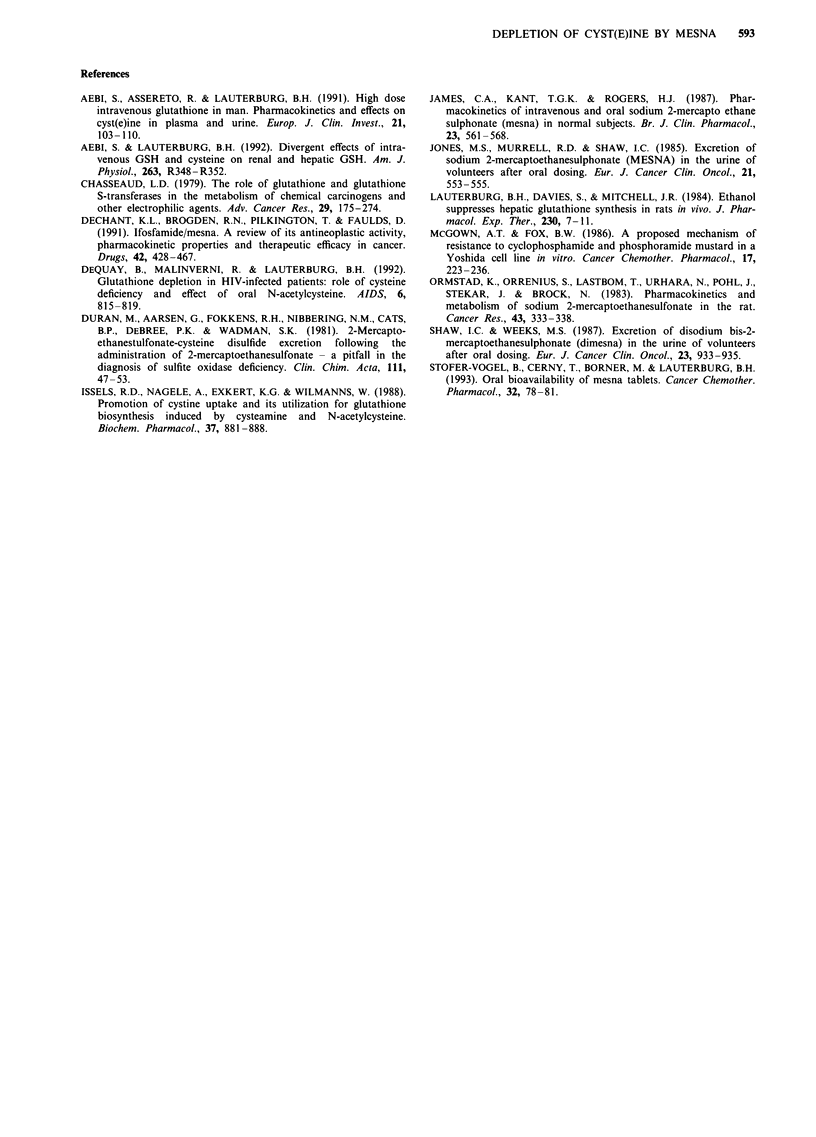# Depletion of circulating cyst(e)ine by oral and intravenous mesna.

**DOI:** 10.1038/bjc.1993.391

**Published:** 1993-09

**Authors:** B. Stofer-Vogel, T. Cerny, A. Küpfer, E. Junker, B. H. Lauterburg

**Affiliations:** Department of Clinical Pharmacology, University of Bern, Switzerland.

## Abstract

The sulfhydryl status of normal and tumour cells is critically important in determining their susceptibility to various cytostatic agents. As a sulfhydryl compound, mesna (sodium 2-mercaptoethane-sulfonate) which is used in large doses to prevent haemorrhagic cystitis associated with certain chemotherapeutic regimens might derange cellular thiol homeostasis. In order to investigate the effects of mesna on the concentrations of thiols in plasma, cysteine, glutathione and their disulfides were measured by HPLC following the oral and intravenous administration of mesna to healthy volunteers. After 7.3 mmol mesna i.v. free cysteine rose from 8.2 (95% CI 7.0-9.4) nmol ml-1 to 53.6 (47.4-59.8) nmol ml-1 at 5 min, most likely due to reduction of circulating cystine by the sulfhydryl drug. This initial rise was followed by a marked decrease of total cyst(e)ine in plasma from 276 (215-337) nmol ml-1 to a nadir of 102 (89-115) nmol ml-1 between 30-120 min after infusion, most likely due to an increased uptake of cysteine into cells and an increased urinary excretion of cyst(e)ine. Qualitatively similar changes were seen after oral mesna. The present data indicate that mesna depletes circulating cyst(e)ine and may thereby markedly alter the sulfhydryl status of cells in vivo although the drug itself is not taken up by most cells.


Br. J. Cancer (1993), 68, 590-593 © Macmillan Press Ltd., 1993

### Depletion of circulating cyst(e)ine by oral and intravenous mesna

B. Stofer-Vogel, T. Cerny, A. Küpfer, E. Junker & B.H. Lauterburg

*Department of Clinical Pharmacology, University of Bern and Institute of Medical Oncology, Inselspital, Bern, Switzerland.*

**Summary** The sulfhydryl status of normal and tumour cells is critically important in determining their susceptibility to various cytostatic agents. As a sulfhydryl compound, mesna (sodium 2-mercaptoethane-sulfonate) which is used in large doses to prevent haemorrhagic cystitis associated with certain chemotherapeutic regimens might derange cellular thiol homeostasis. In order to investigate the effects of mesna on the concentrations of thiols in plasma, cysteine, glutathione and their disulfides were measured by HPLC following the oral and intravenous administration of mesna to healthy volunteers. After 7.3 mmol mesna i.v. free cysteine rose from 8.2 (95% CI 7.0–9.4) nmol ml$^{-1}$ to 53.6 (47.4–59.8) nmol ml$^{-1}$ at 5 min, most likely due to reduction of circulating cystine by the sulfhydryl drug. This initial rise was followed by a marked decrease of total cyst(e)ine in plasma from 276 (215–337) nmol ml$^{-1}$ to a nadir of 102 (89–115) nmol ml$^{-1}$ between 30–120 min after infusion, most likely due to an increased uptake of cysteine into cells and an increased urinary excretion of cyst(e)ine. Qualitatively similar changes were seen after oral mesna. The present data indicate that mesna depletes circulating cyst(e)ine and may thereby markedly alter the sulfhydryl status of cells *in vivo* although the drug itself is not taken up by most cells.

The sulfhydryl status of tumour cells is critically important in determining their susceptibility to various cytostatic agents (McGown & Fox, 1986). Similarly, the sulfhydryl status of normal cells is an important determinant of their defense against the toxic effects of the same cytostatic drugs (Chasseaud, 1979). Mesna (sodium 2-mercaptoethane-sulfonate) is increasingly used to prevent haemorrhagic cystitis associated with chemotherapeutic regimens containing high doses of ifosfamide and cyclophosphamide (Dechant *et al.*, 1991). Although mesna is very polar and does not passively enter cells to a significant extent (Ormstad *et al.*, 1983), the sulfhydryl drug could still markedly affect cellular thiol homeostasis. High concentrations of the sulfhydryl could reduce circulating cystine, thereby making more cysteine available to cells since cysteine is more readily taken up by many cell types than cystine (Issels *et al.*, 1988). In addition, cysteine-mesna mixed disulfides have been identified in urine (Jones *et al.*, 1985; Duran *et al.*, 1981). Mesna might thus result in a substantial loss of cyst(e)ine. Since the effects of intravenous and oral mesna on circulating physiological thiols are not known and the mesna-induced loss of cyst(e)ine has not been quantitated, the concentrations of cysteine, glutathione and their disulfides in plasma and urine were measured following the oral and intravenous administration of mesna to healthy volunteers.

### Subjects and methods

The effects of mesna (sodium-2-mercaptoethane-sulfonate) on plasma cyst(e)ine and glutathione after intravenous and oral administration were studied in eight healthy volunteers, two females and six men, 24 to 39 years of age, all within 10% of ideal body weight. Informed consent was obtained from each of the participants. The study was approved by the ethics committee of the local medical school. After an overnight fast an indwelling catheter was placed into an antecubital vein of both arms in order to obtain blood repeatedly without tourniquet. Two blood samples were obtained 10 min apart before the administration of mesna in order to determine the basal concentrations of glutathione and cyst(e)ine.

Intravenous mesna was infused over 2 min at a dose of 1.2 g (7.3 mmol; ASTA MEDICA, 400 mg ml$^{-1}$) and blood samples were obtained 0, 5, 10, 15, 30, 60, 120, 240 min after termination of the infusion. Urine was collected before and during the 4 h after administration of mesna. In two patients peripheral blood mononuclear cells were isolated using Ficoll-Paque at baseline and 30 and 120 min following the infusion of mesna and were processed as previously described for the determination of intracellular cysteine (DeQuay *et al.*, 1992).

Since much lower plasma concentrations of free mesna are seen after oral administration (James *et al.*, 1987) the effect of oral mesna on the thiol status was also investigated. Mesna was administered at a dose of 1.2 g (ASTA MEDICA, four tablets of 300 mg) together with 200 ml of water and blood was collected after 0.5, 1, 1.5, 2, 2.5, 3, 4, 6 and 8 h. Urine was collected before ingestion of mesna and at intervals thereafter for 8 h. Food and liquids were allowed 2 h after the administration of mesna.

### *Analytical methods*

Five ml of blood were collected into heparinised tubes containing L-serine-Na-borate (final concentration 2 mmol l$^{-1}$) in order to prevent the degradation of glutathione by gamma-glutamyltransferase and were immediately centrifuged. Within 2 min of collection plasma was derivatised with monobromobimane (10 $\mu$l of a solution of 25 mmol l$^{-1}$ in acetoninitrile; Thiolite reagent, Calbiochem, La Jolla, USA). D-penicillamine served as internal standard. Calibration curves were established daily by adding known amounts of mesna, cysteine and glutathione to plasma samples (Stofer *et al.*, 1993).

Total mesna, cyst(e)ine and glutathione, i.e. free thiols, thiol disulfides and small molecular and protein mixed disulfides, were measured after reduction of disulfides with dithiothreitol (Aebi *et al.*, 1991). Total mesna and cyst(e)ine in urine were measured following the same protocol as for disulfides in plasma. Chromatography and quantification of sulfhydryls were performed as previously described (Stofer *et al.*, 1993). Taking a blood sample five times through the procedure the coefficients of variation for total mesna, cyst(e)ine and GSH were 9.8, 1.6 and 3.0%, respectively. The coefficients of variation for the corresponding free sulfhydrils were <5%. The recovery of mesna, cystine and glutathione disulfide added to plasma was 102 ± 10%, 105 ± 8% and 85 ± 9% (mean ± s.d., $n = 5$), respectively. The peak to noise ratio for concentrations of cysteine and GSH of 5 $\mu$mol l$^{-1}$ exceeds five.

### *Data analysis*

The results are given as mean and 95% confidence intervals.

Correspondence: B.H. Lauterburg, Department of Clinical Pharmacology, Murtenstrasse 35, 3010 Bern, Switzerland.
Received 1 March 1993; and in revised form 29 April 1993.

### Results

After intravenous administration the plasma concentration of mesna decreased from a peak of 511 (95% CI 404–617) nmol ml$^{-1}$ at 5 min with a half life of 19 (17–21) min. After oral administration peak concentrations of 33 (26–40) nmol ml$^{-1}$ were reached between 1.5 and 4 h after ingestion. Peak concentrations of total mesna, i.e. free mesna and its disulfides, averaged 820 and 139 nmol ml$^{-1}$ following intravenous and oral administration, respectively (Stofer *et al.*, 1993).

As shown in Figure 1 intravenous mesna resulted in a marked increase in circulating free cysteine from 8.2 (95% CI 7.0–9.4) nmol ml$^{-1}$ to 52.5 (44.1–61.0) nmol ml$^{-1}$ 5 min after the end of the infusion, suggesting that mesna reduces circulating cystine. In contrast to the early transient increase in free cysteine total cyst(e)ine decreased from its basal concentration of 276 (215–337) nmol ml$^{-1}$ to a nadir of 102 (89–115) nmol ml$^{-1}$ between 30–120 min after infusion. The concentrations gradually returned to 198 (165–230) nmol ml$^{-1}$ at 4 h. Following oral administration of mesna a small increase in free cysteine was again observed (Figure 2). Total cyst(e)ine decreased from 241 (216–267) nmol ml$^{-1}$ to a nadir of 120 (97–142) nmol ml$^{-1}$ and had not returned to the basal level by 8 h. There was a significant ($P<0.001$) correlation between the increment in free cysteine and the peak concentrations of free ($r^2 = 0.852$) and total ($r^2 = 0.818$) mesna (Figure 3). The decrease in circulating cyst(e)ine showed a significant ($P<0.001$) correlation ($r^2 = 0.579$) with the area under the plasma concentration time curve of free mesna (Figure 3).

In contrast to cystine, cysteine is readily taken up by cells. In order to see whether the mesna-induced increase in circulating free cysteine resulted in an increased intracellular concentration of cysteine peripheral blood mononuclear cells were isolated and their cysteine content was measured. As shown in Figure 4 there was a marked increase in intracellular cysteine 30 min after infusing mesna in the two subjects studied. Intracellular cysteine had returned to baseline by 2 h.

The decrease in circulating cyst(e)ine was associated with a marked increase in the urinary excretion of the amino acid from 40 (33–47) nmol cysteine μmol$^{-1}$ creatinine to 105 (90–120) nmol μmol$^{-1}$ creatinine during the 4 h following the intravenous administration of mesna. Assuming a constant excretion of creatinine throughout the day the mesna-induced increment in the urinary excretion of cysteine averaged 0.26 mmol in 4 h. Following oral administration of mesna the urinary excretion of cyst(e)ine increased to 153 (116–190) between 2–4 h, 120 (96–143) between 4–6 h, and 79 (63–95) nmol cyst(e)ine μmol$^{-1}$ creatinine between 6–8 h

**Figure 1** Plasma concentrations of free cysteine (squares) and total cyst(e)ine (triangles) following intravenous infusion of 1.2 g of mesna (mean + 95% CI, $n = 8$).

**Figure 2** Plasma concentrations of free cysteine (squares) and total cyst(e)ine (triangles) following oral administration of 1.2 g of mesna (mean + 95% CI, $n = 8$).

**Figure 3** Correlation between the peak concentration of free mesna and the increment in cysteine ($r^2 = 0.852$) and between the decrease in circulating total cyst(e)ine and the area under the plasma concentration time curve of free mesna ($r^2 = 0.579$) following oral (open circles) and intravenous (closed circles) administration of mesna. Stippled lines = 95% confidence interval.

**Figure 4** Concentration of cysteine in peripheral blood mononuclear cells isolated from two subjects before and 30 and 120 min after the intravenous administration of mesna.

after oral dosing. The mesna-induced urinary loss of cyst(e)ine during the 8 h following the oral administration amounted to 0.52 (0.42–0.63) mmol.

Following intravenous mesna there was a small rise in circulating free glutathione (Figure 5). No change in free and total glutathione was seen after oral mesna. No glutathione was found in urine.

### Discussion

Mesna had two striking effects on circulating cyst(e)ine: First, it markedly increased circulating free cysteine, and secondly, it resulted in profound depletion of circulating total cyst(e)ine. The effect on free cysteine was much more evident following intravenous administration when markedly higher concentrations of free mesna are achieved than after oral administration. A similar increase in free cysteine has previously been seen after the intravenous administration of large doses of glutathione (Aebi *et al.*, 1991), suggesting that it most likely results from the reduction of cystine by high circulating concentrations of thiols. In contrast to cystine, cysteine is readily taken up by cells (Issels *et al.*, 1988). Thus, rising the concentration of free cysteine in the extracellular space would be expected to increase the intracellular concentration of cysteine. Indeed, in the two subjects where intracellular cysteine was measured there was a transient marked increase in intracellular cysteine (Figure 4).

A shift of cysteine from the extracellular to the intracellular compartment can in part explain the second effect of mesna on circulating cyst(e)ine, namely the marked depletion of total cyst(e)ine which followed the initial rise in free cysteine. In addition, mesna resulted in a marked increase in the urinary excretion of cyst(e)ine. Mesna is in part excreted in the form of mesna-cysteine disulfide and may also circulate as a mixed disulfide (Jones *et al.*, 1985; Duran *et al.*, 1981). This fraction appears to be larger after oral administration such that more cyst(e)ine is lost via the kidneys following oral administration. Free cysteine in urine may contribute to the uroprotective effects of mesna.

Mesna had minimal short-term effects on circulating glutathione. In contrast to cystine, glutathione disulfide and mixed disulfides account for less than 50% of total circulating glutathione. Thus, in the presence of even high concentrations of mesna much less free glutathione than cysteine will be generated.

The quantitative importance and the metabolic consequences of the suggested translocation of cysteine are difficult to assess. Assuming a volume of distribution for total cyst(e)ine corresponding to the extracellular space the observed depletion of cyst(e)ine 1 h following intravenous mesna corresponds to an estimated disappearance of 1–2 mmol of cyst(e)ine. Since the increment in the urinary excretion of cyst(e)ine during the first 4 h amounted to only about one fourth of this amount substantial quantities of cysteine must have been translocated into cells.

Since the availability of cysteine is rate-limiting for the synthesis of glutathione *in vivo* (Aebi *et al.*, 1992), translocation of extracellular cysteine into the intracellular compartment might result in an increased synthesis of glutathione which plays an important role in the detoxification of reactive metabolites of cytostatic agents and other compounds. On the other hand, the turnover of cysteine is very rapid (Lauterburg *et al.*, 1984), such that a substantial fraction of the increased cellular content of cysteine may be utilised by pathways other than glutathione synthesis. Such an increase in the catabolism of cysteine might eventually decrease the availability of cysteine and lead to depletion of glutathione. Preliminary results of an ongoing study in patients who receive mesna together with ifosfamide by continuous infusion show a marked depletion of circulating cyst(e)ine, glutathione and homocysteine, indicating that similar changes in sulfhydryl homeostatis occur during chemotherapy.

The present data indicate that the reaction of the free sulfhydril group of mesna with endogenous disulfides is probably mainly responsible for the mesna-induced disruption of thiol homeostasis. Since oral mesna increases circulating free cysteine to a lesser extent than intravenous mesna and leads to a more marked increase in urinary cyst(e)ine which may contribute to uroprotection, oral administration of mesna may be preferable. In view of the fact that the efficacy of mesna critically depends on the reduction by the kidneys of mesna-mixed disulfides and dimesna a case could be made for the therapeutic use of dimesna which exhibits comparable uroprotective properties as mesna (Shaw & Weeks, 1987) but is not likely to interfere with thiol homeostasis to the same extent as mesna.

**Figure 5** Plasma concentrations of free glutathione following intravenous infusion of 1.2 g of mesna (mean + 95% CI, $n = 8$).

Supported by grant # 32-29943.90 from the Swiss National Foundation for Scientific Research and ASTA Medica.

### References

AEBI, S., ASSERETO, R. & LAUTERBURG, B.H. (1991). High dose intravenous glutathione in man. Pharmacokinetics and effects on cyst(e)ine in plasma and urine. *Europ. J. Clin. Invest.*, **21**, 103–110.

AEBI, S. & LAUTERBURG, B.H. (1992). Divergent effects of intravenous GSH and cysteine on renal and hepatic GSH. *Am. J. Physiol.*, **263**, R348–R352.

CHASSEAUD, L.D. (1979). The role of glutathione and glutathione S-transferases in the metabolism of chemical carcinogens and other electrophilic agents. *Adv. Cancer Res.*, **29**, 175–274.

DECHANT, K.L., BROGDEN, R.N., PILKINGTON, T. & FAULDS, D. (1991). Ifosfamide/mesna. A review of its antineoplastic activity, pharmacokinetic properties and therapeutic efficacy in cancer. *Drugs*, **42**, 428–467.

DEQUAY, B., MALINVERNI, R. & LAUTERBURG, B.H. (1992). Glutathione depletion in HIV-infected patients: role of cysteine deficiency and effect of oral N-acetylcysteine. *AIDS*, **6**, 815–819.

DURAN, M., AARSEN, G., FOKKENS, R.H., NIBBERING, N.M., CATS, B.P., DEBREE, P.K. & WADMAN, S.K. (1981). 2-Mercapto-ethanestulfonate-cysteine disulfide excretion following the administration of 2-mercaptoethanesulfonate – a pitfall in the diagnosis of sulfite oxidase deficiency. *Clin. Chim. Acta*, **111**, 47–53.

ISSELS, R.D., NAGELE, A., EXKERT, K.G. & WILMANNS, W. (1988). Promotion of cystine uptake and its utilization for glutathione biosynthesis induced by cysteamine and N-acetylcysteine. *Biochem. Pharmacol.*, **37**, 881–888.

JAMES, C.A., KANT, T.G.K. & ROGERS, H.J. (1987). Pharmacokinetics of intravenous and oral sodium 2-mercapto ethane sulphonate (mesna) in normal subjects. *Br. J. Clin. Pharmacol.*, **23**, 561–568.

JONES, M.S., MURRELL, R.D. & SHAW, I.C. (1985). Excretion of sodium 2-mercaptoethanesulphonate (MESNA) in the urine of volunteers after oral dosing. *Eur. J. Cancer Clin. Oncol.*, **21**, 553–555.

LAUTERBURG, B.H., DAVIES, S., & MITCHELL, J.R. (1984). Ethanol suppresses hepatic glutathione synthesis in rats *in vivo*. *J. Pharmacol. Exp. Ther.*, **230**, 7–11.

MCGOWN, A.T. & FOX, B.W. (1986). A proposed mechanism of resistance to cyclophosphamide and phosphoramide mustard in a Yoshida cell line *in vitro*. *Cancer Chemother. Pharmacol.*, **17**, 223–236.

ORMSTAD, K., ORRENIUS, S., LASTBOM, T., URHARA, N., POHL, J., STEKAR, J. & BROCK, N. (1983). Pharmacokinetics and metabolism of sodium 2-mercaptoethanesulfonate in the rat. *Cancer Res.*, **43**, 333–338.

SHAW, I.C. & WEEKS, M.S. (1987). Excretion of disodium bis-2-mercaptoethanesulphonate (dimesna) in the urine of volunteers after oral dosing. *Eur. J. Cancer Clin. Oncol.*, **23**, 933–935.

STOFER-VOGEL, B., CERNY, T., BORNER, M. & LAUTERBURG, B.H. (1993). Oral bioavailability of mesna tablets. *Cancer Chemother. Pharmacol.*, **32**, 78–81.